# INs and OUTs of faces in consciousness: a study of the temporal evolution of consciousness of faces during binocular rivalry

**DOI:** 10.3389/fnhum.2023.1145653

**Published:** 2023-05-22

**Authors:** Thomas Quettier, Nicolò Di Lello, Naotsugu Tsuchiya, Paola Sessa

**Affiliations:** ^1^Department of Developmental and Social Psychology, University of Padova, Padua, Italy; ^2^Padova Neuroscience Center (PNC), University of Padova, Padua, Italy; ^3^Turner Institute for Brain and Mental Health & School of Psychological Sciences, Faculty of Medicine, Nursing, and Health Sciences, Monash University, Melbourne, VIC, Australia; ^4^Center for Information and Neural Networks (CiNet), National Institute of Information and Communications Technology (NICT), Osaka, Japan; ^5^Advanced Telecommunications Research Computational Neuroscience Laboratories, Kyoto, Japan

**Keywords:** binocular rivalry, consciousness time course, facial expression, happy expressions, joystick

## Abstract

Contents of consciousness change over time. However, the study of dynamics in consciousness has been largely neglected. Aru and Bachmann have recently brought to the attention of scientists dealing with consciousness the relevance of making inquiries about its temporal evolution. Importantly, they also pointed out several experimental questions as guidelines for researchers interested in studying the temporal evolution of consciousness, including the phases of formation and dissolution of content. They also suggested that these two phases could be characterized by asymmetric inertia. The main objective of the present investigation was to approximate the dynamics of these two phases in the context of conscious face perception. To this aim, we tested the time course of content transitions during a binocular rivalry task using face stimuli and asked participants to map their subjective experience of transitions from one content to the other through a joystick. We then computed metrics of joystick velocity linked to content transitions as proxies of the formation and dissolution phases. We found a general phase effect such that the formation phase was slower than the dissolution phase. Furthermore, we observed an effect specific to happy facial expressions, such that their contents were slower to form and dissolve than that of neutral expressions. We further propose to include a third phase of stabilization of conscious content between formation and dissolution.

## Introduction

Contents of consciousness change over time. These transitions from one content to another content characterize our whole mental life, from perceptions in the different sensory modalities (e.g., as it happens during the succession of words in consciousness during reading) to thoughts (e.g., when thinking back to the points of a to-do list of the day), and are definitely shaped by attentional and memory processes. Despite this intuition granted on our subjective experience of transitioning from one conscious content to another content, scientific research has largely neglected the study of how these changes occur and evolve over time.

In the present investigation, we sought to examine transitions of consciousness contents that could be reliably monitored in a laboratory setting by focusing on the transitions of contents of visual stimuli presented in binocular rivalry (BR; Wheatstone, 1843).

**FIGURE 1:**
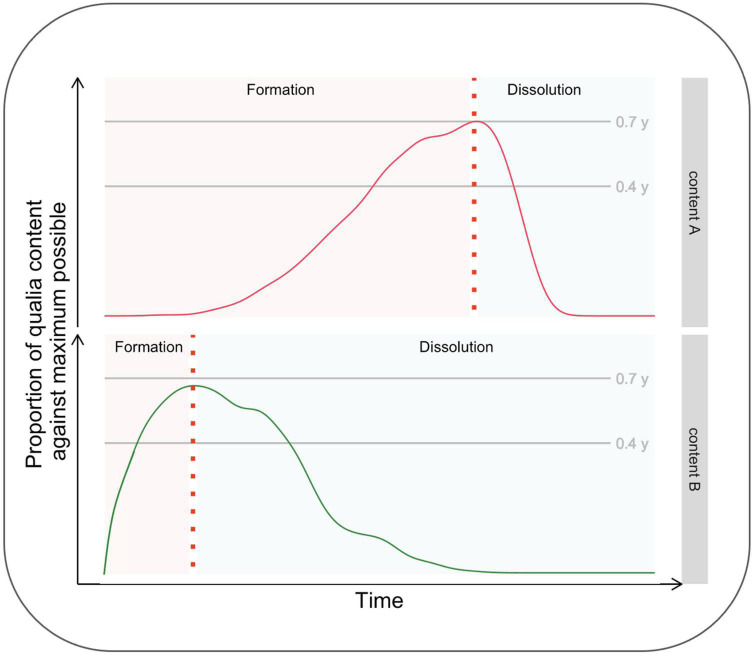
Hypothetical time course of the formation and dissolution of two different contents, A and B.

Aru and Bachmann (2017) have recently brought to the attention of scientists dealing with consciousness the relevance of making inquiries about its temporal evolution. While this opinion piece has not received much citation, it is one of Frontiers’ most viewed articles,^1^ thus suggesting significant interest by the scientific community regarding this topic. The kind of investigation suggested by these authors is all the more relevant when considering a micro-genetic tradition, according to which the *formation* of conscious content is not instantaneous but, indeed, time-consuming. From a phenomenological perspective, the content enriches over time by acquiring a more significant number of qualities, becoming clearer, more stable, and more detailed. Similarly, Aru and Bachmann (2017) proposed that investigating the phase of *dissolution* from consciousness, what they define as the “anti-genesis” of consciousness, is equally crucial. Importantly, they also point out several experimental questions as guidelines for researchers interested in studying the temporal evolution of consciousness, including the phases of formation and dissolution of a content. For example, whether the formation and dissolution phases’ inertia is symmetric (or not) is unknown. It is also unknown if the inertia of the two phases varies as a function of stimuli parameters (such as contrast) in a way that their time course can be manipulated by the experimenters. Figure 1 shows the dynamics of “conscious experience evolving over time” (Aru and Bachmann, 2017; Figure 1, p. 2) for two different conscious contents, A and B, differing in terms of formation and dissolution time-courses.

To summarize, Aru and Bachmann stressed the benefit of investigating both the formation and dissolution phases and considered the possibility that these two phases do not have the same duration and that stimuli of different natures may be characterized by differences in the duration of the two phases.

In the present work, we present a method to investigate the dynamics of conscious experience of faces characterized by an emotional expression or gender. Here, the “dissolution” and “formation” phases are not meant to imply that something happens prior to the perceptual change, but rather they indicate the visual perceptual changes (as experienced by the subjects) themselves.

We implemented a variant of a binocular rivalry (BR) paradigm. Under ecological circumstances, slightly discordant visual inputs to the two eyes result in a stable stereo experience. When the dissimilarity exceeds a certain threshold, periods of perceptual dominance of one stimulus over the other stochastically alternate, such that one monocular image is dominantly consciously experienced while the other is suppressed and invisible (Alais and Blake, 2005; Brascamp et al., 2015). This is the condition called BR; phenomenologically, the visual *quale* of one stimulus comes in and goes out over time. According to the theories of consciousness, the shift from unconscious to conscious perception arises as a process of formation; as such, the *formation phase* consists in updating the current version of the phenomenal content (Bachmann, 2000; Aru and Bachmann, 2017), i.e., conscious content arises and replaces the previous content. To note, BR conscious content is also often the result of the integration of processing from multiple systems (e.g., visual and proprioceptive), as we showed in one previous study using BR with facial expressions (Quettier et al., 2021). As such, during the formation phase, processing from multiple channels/brain regions is integrated to generate that particular conscious content and no other content. This might suggest that the processing of stimuli that varies on the number of involved channels/brain regions could lead to differences in the inertia of the formation and/or dissolution phases. Changes in visual experience during formation and/or dissolution phases can occur in different ways, such as through traveling wave-like transitions or local dis/appearing, as described by Yang et al. (1992) and Lee et al. (2005).

When using a simple two-choice response (A or B content) to monitor participants’ experience of alternations in BR, the researcher can only analyze data about the onset of consciousness (formation phase; Aru and Bachmann, 2017) and data about the stabilization/maintenance of the conscious content (Quettier et al., 2021). On the other hand, some studies have employed a three-choice response procedure (including a response for the experience of something unclear, i.e., “mixed” percept”; Knapen et al., 2011; Davidson et al., 2018). However, this procedure too, although providing a higher resolution of participants’ subjective experience during the BR, cannot characterize the phases of formation and dissolution in a fine-grained way. Skerswetat and Bex (2023) provided a detailed historical overview of methods in reporting subjective experience in BR.

Here we asked participants to map their subjective experience of transitions in BR using a joystick. The rationale behind this methodological choice is that the time course of joystick responses can be considered a good proxy for the dynamics of “conscious experience evolving over time” as proposed by Aru and Bachmann (Naber et al., 2011). Despite criticisms of the use of a joystick as a reporting tool (Fahle et al., 2011), we assumed that delay in the participants’ report with respect to the subjective expeience would be consistent across conditions and thus would not have a significant impact on our results.

To analyze the time-series data of BR obtained using the joystick, we defined a few dependent variables to estimate the inertia of the formation and dissolution phases and the periods of stabilization of contents:

1. kinematic speed parameters able to capture the BR transitions as measured by the joystick movements, i.e., *speed* (|*V|*) that could be considered as a proxy of the time-course of the formation (Speed_IN_; |*V| -in*) and dissolution (Speed_OUT_; |*V| -out*) phases of conscious contents (see Methods for details);
2. a measure of the proportion of total time of dominance in awareness of one percept over the other during the same rival competition, excluding the periods of transitions and the initial competition, i.e., *cumulative time* (*CT*). In other words, CT is a metric that allows an estimate of the periods of stable resolution following the initial competition (see Alpers and Gerdes, 2007) for similar measures);
3. a measure of the proportion of total time of dominance in awareness of one percept over the other during the same rival competition, including the time needed for the initial disambiguation and the periods of transitions, i.e., *mean of predominance* (*MP*). In other words, *MP* is a metric that estimates the periods of relative preference of one percept over the other.

We have shared all the necessary information, including scripts and a step-by-step description of the procedure to compute the metrics presented above here: https://osf.io/2pzmg/.

We have implemented two BR conditions, a “facial expression rivalry” and a “gender rivalry”. In the first condition, the stimuli placed in rivalry were two different facial expressions of the same individual (neutral vs. happy); in the second condition, the rivalrous stimuli were the faces of individuals of different gender (female vs. male) but with the same expression.

The main objective of this work was to approximate the temporal evolution of consciousness for faces characterized by specific attributes (emotional expression and gender).

We had no precise predictions regarding the speed of the formation and dissolution phases for the different categories of stimuli, although, as regards the “facial expression rivalry”, several studies using the standard two/three-choice response approach have shown that happy expressions dominate in awareness and are associated with longer stabilization times (i.e., cumulative time) (Alpers and Gerdes, 2007; Bannerman et al., 2008; Yoon et al., 2009; Quettier et al., 2021). As briefly discussed in a previous paragraph, these response methods do not allow monitoring the entire temporal evolution of content in awareness. Nonetheless, higher dominance rates for faces with happy vs. neutral expressions may suggest a longer formation and/or dissolution phase duration for the former than the latter.

## Materials and methods

*Participants.* Based on multiple findings in BR, happy stimuli rivaling versus neutral stimuli lead to large effect sizes (Alpers and Gerdes, 2007; Yoon et al., 2009; Hernández-Lorca et al., 2019; Quettier et al., 2021). For medium effect size (i.e *d* = 0.5), a sample size of 34 participants is required to reach an 80% power level. Power has been estimated using the pwr package (Champely et al., 2017). Forty healthy participants were recruited among students at the University of Padua (average age in years = 22.35, SD = 2.6, 20 males, 3 left-handed). All of them were volunteers and gave written informed consent in accordance with the Declaration of Helsinki. All experimental procedures were previously approved by the local research ethics committee (Comitato Etico della Ricerca Psicologica Area 17, University of Padua) and performed in accordance with its guidelines. Participants self-reported to have a normal or corrected-to-normal vision. Because we administered stimuli with emotional content, participants completed the Toronto Alexithymia Scale (TAS-20) and the Interpersonal Reactivity Index (IRI) questionnaires at the end of the experiment to obtain indices, however crude, that could reassure us about a normotypical affective competence. Alexithymia is defined as a difficulty in experiencing emotions, and Empathy is defined as the ability to share and understand others’ emotions and affective states (Nemiah et al., 1976; Zaki and Ochsner, 2012). Scores on both questionnaires were not in the cut off range (TAS-20: *M* = 46.05, SD = 12.12 (Parker et al., 2003); IRI: *M* = 100.2, SD = 10.45 (Maddaluno et al., 2022)).

*Material and apparatus.* Visual stimuli were displayed using Opensesame v 3.0 on a Bestview S5 (luminosity: 50; contrast: 50) 60 Hz monitor mounted on a stereoscope mounted on a chin-rest. Visual stimuli covered 6.5 ± 0.5 degrees of visual angle and 10 cm in height and width. Original pictures (AM10NES, AM10HAS, AF01NES, AF01HAS) of facial expressions have been selected from the Karolinska Directed Emotional Faces set (KDEF).^2^ A white 12-pixel fixation point and 40-pixel black and white squares frame were applied to the images to facilitate binocular fusion using GIMP (version 2.8.10).^3^ Monocular images contrast and luminance features were controlled (Skerswetat et al., 2018) by matching histograms by using Fiji (ImageJ 1.52c)^4^ and faces where misaligned in opposite direction with respect to the fixation point by 4 pixels.

**FIGURE 2:**
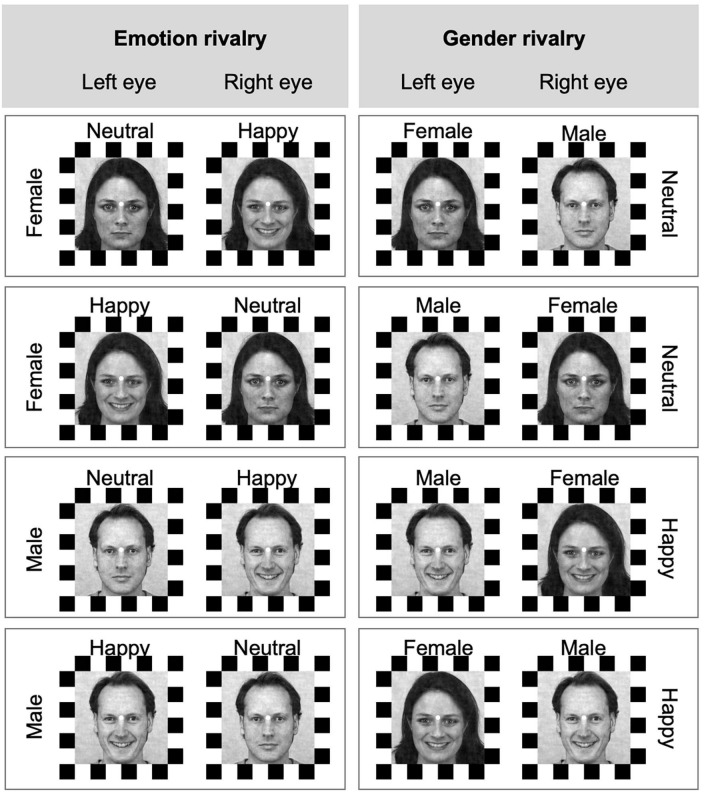
Rival conditions for expression and gender rivalries. Each row represents a pair of stimuli presented in rivalry. The four possible combinations of stimulus and eye of presentation are presented in each column. In the expression rivalry **(left column)**, the same identity is presented during a trial, and the expressions are in competition. In the gender rivalry **(right column)**, the same expression is presented during a trial, and the identities are in competition. Face stimuli from the Karolinska Directed Emotional Faces can be freely used for non-commercial research purposes. We included information about the KDEF images’ ID in the main text.

*Procedure.* Participants were seated on a comfortable chair in a silent, temperature-controlled room. Before starting the experiment, the visual apparatus was set, and participants were trained. During the training, participants reported the rivalry experience by using both joystick and speech to ensure they undertood the task instructions. During the experiment, participants were asked to focus on a fixation point placed in the middle of the visual field. The experiment consisted of one session of four blocks, with two blocks for “emotion rivalry” (happy vs. neutral facial expression rivalry, Figure 1 left) and two blocks for “gender rivalry” (male vs. female face gender rivalry, Figure 1 right). The order of these emotional expressions or gender rivalry conditions was counterbalanced between subjects. In each block, combinations of rivalry stimuli (i.e., 4 pairs; see Figure 2) were randomly presented three times, for a total of 12 trials in each block (for a total of 48 trials in the experiment). See Figure 2, which shows all the possible combinations of rivalry stimuli shown in the experiment.

Each stimulus was presented for 15 s, preceded by a 2-s fixation point, followed by a black screen lasting 3 s, we avoided long BR exposure to minimize mixed perception (Klink et al., 2010). Participants were informed that, on each trial, they could see one of two faces and that the appearance might change from one to the other during the trial. Participants were asked to report their visual experience in real-time by means of a joystick over the left-right axis range. The trial started with a central position of the joystick, with the leftmost and rightmost positions corresponding to the stimulus “clearly” seen according to the coding instruction. Coding instructions were presented before the beginning of the block; the order of the “left” and “right” joystick positions corresponding to the coding of the “happy or male” and “neutral or female” faces was counterbalanced across blocks. Joystick responses were recorded at 100 Hz sampling frequency. At the end of each block, a short break was recommended to the participants to avoid any kind of visual tiredness. At the end of the last block, valence and arousal of each stimulus were measured respectively on a -3/ + 3 and 1/7 Likert scales using custom keyboard keys. Valence and arousal evaluation may be an important control to ensure that participants assigned aan emotional meaning to happy faces when compared to neutral faces (Hodges and Fox, 1965; Carter et al., 2007).

### Data reduction

*Preprocessing.* Joystick positions were a continuous signal ranging from -1 (i.e. leftmost position) to 1 (i.e., rightmost position), or vice-versa according to the counterbalance. All trials were aligned, multiplying by -1 those of which the range was from 1 to −1.

**FIGURE 3:**
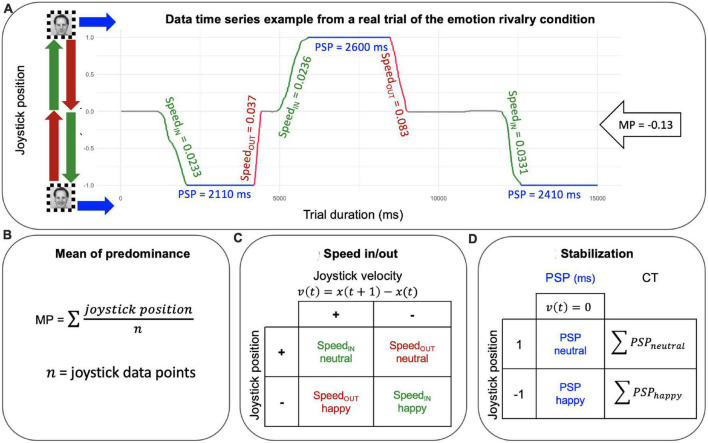
**(A)** Joystick time series from a real trial, which is one of 12 unique trials collected from one participant and serves as an example to illustrate how the measures are applied to the data. The green segments represent SpeedIN, which is the period of joystick movement as it transitions into a new perceptual state. The red segments represent SpeedOUT, which is the period of joystick movement as it exits from the dominant content. The blue segments represent periods of stable perception (PSP), during which a single perceptual state is dominant and the joystick is stationary. Joystick position and time are projected on the x and y axes, respectively. **(B)** Mean of predominance is the mean of all joystick position data points. **(C)** Speed movements were extracted from trials by averaging the absolute velocity separately for the formation (SpeedIN) and the dissolution (SpeedOUT) epochs and for each percept within each rivalry condition (happy and neutral for the emotion rivalry, and male and female for gender rivalry). **(D)** Cumulative time (CT) was computed as the sum of PSPs (i.e. when the joystick position was 1/-1, that is velocity was equal to zero) for each percept separately for each rivalry condition (happy and neutral for the emotion rivalry, and male and female for gender rivalry).

*Postprocessing.* We computed three measures from the data time series: (1) the mean of predominance (MP) as a measure of perceptual dominance. MP is a measure of the dominance between the two stimuli in rivalry (happy vs. neutral expressions OR male vs. female). MPs were computed by averaging the joystick signals for each trial and each participant; (2) cumulative time (CT), as a measure of the BR stabilization (i.e., the sum of PSPs epochs). Cumulative time (CT) was computed as the sum of periods of stable perception (i.e. when the joystick position was 1/-1, that is, velocity was equal to zero) for each percept separately for each rivalry condition (happy and neutral for the emotion rivalry, and male and female for gender rivalry). (3) Speed_IN_ and Speed_OUT_ as measures of the BR transitions. The speed of the joystick was estimated by averaging the absolute velocity separately for each episode of the formation (Speed_IN,_ Figure 3A green lines) and the dissolution (Speed_OUT,_ Figure 3A red lines) for each percept within each rivalry condition (happy and neutral for the emotion rivalry, and male and female for gender rivalry; see Figure 2).

### Data analysis

Differences in stimuli rating for valence and arousal and differences in rivalry conditions (emotion rivalry and gender rivalry) for MPs were assessed in separate analyses of variance (ANOVAs) and *post hoc* comparisons (paired t.test) since we had no specific *a priori* predictions. All *post hoc* comparisons were corrected for multiple comparisons (Bonferroni), to ensure that the cumulative Type I error was below.05. We used R (R Core Team, 2012) and lme4 (Bates et al., 2014) to perform a linear mixed effects analysis of the relationship between rivalry conditions (emotion or gender) and consciousness phases (Speed_IN_ and Speed_OUT_) for cumulative time (CT). For all models, as random effects, we had intercepts for subjects. Visual inspection of residual plots did not reveal any obvious deviations from homoscedasticity or normality. Model selection is based on the likelihood of different models’ comparison (see Open science framework repository).^5^ For CT models, as fixed effects, we entered rivalry (i.e., emotion rivalry or gender rivalry) into the model. *P*-values were obtained by ANOVA of the full models for emotion blocks: *CT*∼ *rivalry* + *(1*| *subject)* and null model for gender *CT*∼ *1* + *(1*| *subject)*. For Speed_IN_ and Speed_OUT_ models, as fixed effects, we entered rivalry and phases (with interaction term) into the model. P-values were obtained by ANOVA of the full models for emotion blocks: *speed*∼ rivalry* phase + *(1*| *subject)* and model 2 for gender block: *speed*∼ *phase* + *(1*| *subject).*

## Results

### Ratings

**FIGURE 4:**
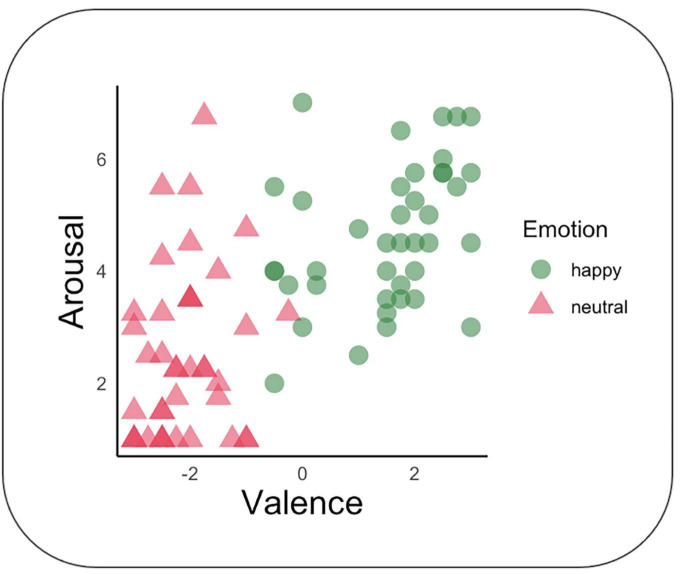
Evaluation of stimuli arousal and valence. Valence and arousal of each stimulus were measured respectively on a -3/+3 and 1/7 scales. Each participant is represented by one circle and one triangle; dark color intensity means that more than one participant gave the same evaluation.

Evaluation of valence and arousal were performed on gender and facial expression stimuli. Valence ratings differed according to *a priori* expectations, *F*(1, 38) = 323.92, *p* < 0.001, *d* = 5.84. Neutral facial expressions were rated under zero (*M* = −2.11; SD = 0.86; range = −3 to 3), which is more negative than happy (*M* = 1.51; SD = 1.29; range = −3 to 3). Arousal ratings also differed according to *a priori* expectations, *F*(1, 39) = 80.45, *p* < 0.001, *d* = 2.91. The ratings were lower for neutral expressions (*M* = 2.49; SD = 1.66; range = 1 to 7) than for happy expressions (*M* = 4.64, SD = 1.36; range = 1 to 7). See Figure 4. Some participants reported spontaneously that the female’s happy facial expression did not appear genuine. No correlations were significant with MP or Speed measures.

### Mean of predominance (MP)

**FIGURE 5:**
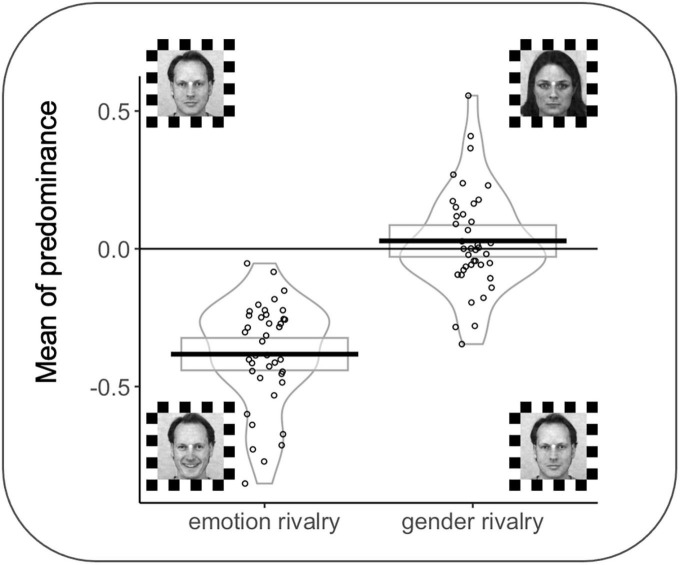
Rectangles, beans, and points represent confidence intervals, smoothed densities, and raw data, respectively. MP = 0 indicates no preference between the two percepts in rivalry during the trial. MPs were computed by averaging the joystick signals for each trial and each participant. Face stimuli from the Karolinska Directed Emotional Faces can be freely used for non-commercial research purposes. We included information about the KDEF images’ ID in the main text.

A significant effect was observed for MP as a function of the rivalry condition (*F*(1,38) = 100.03 *p* < 0.001); interestingly, all participants in the emotion rivalry showed an advantage for the happy expression (vs. neutral expressions). MP for the emotion rivalry in favor of happy faces (*M* = −0.38; SD = 0.19 s) was significantly different from zero, *t*(39) = −12.76, *p* < 0.001. MP for the gender rivalry (*M* = −0.028; SD = 0.17 s) was not significantly different from zero, *t*(39) = 0.967, *p* = 0.339 (see Figure 5).

### Cumulative time (CT)

**FIGURE 6:**
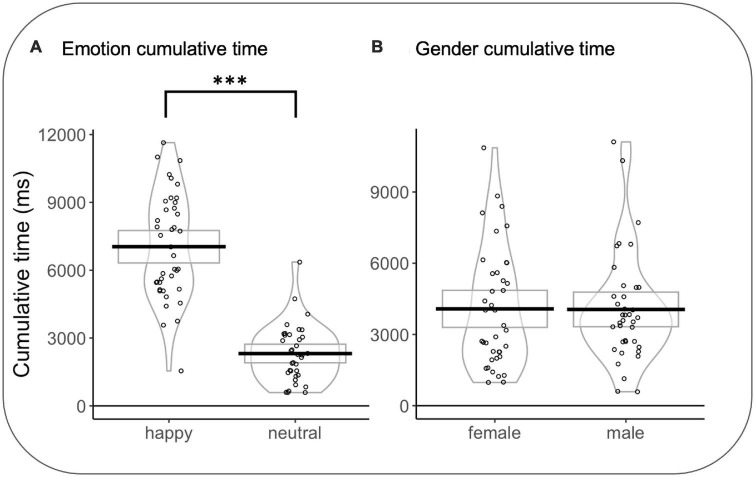
**(A)** Cumulative time in the emotion rivalry condition. **(B)** Cumulative time in gender rivalry condition. Rectangles, beans, and points represent confidence intervals, smoothed densities, and participants’ mean data, respectively. *p<0.05, **p<0.01, ***p<0.001.

In emotion rivalry, a significant effect was observed for CT as a function of the type of emotion (CT_HAPPY_ vs. CT_NEUTRAL_) (*F*(1,635.54) = 407.072, *p* < 0.001, *d* = 1.6): CT_HAPPY_ were longer than CT_NEUTRAL_ *t*(35) = 11.362 *p* < 0.0001, *d* = 1.92). As the better model for gender rivalry is the null model, no effects are considered (see Figure 6).

*Speed*_IN_ *and Speed*_OUT_. In emotion rivalry, a significant effect was observed for the transition (Speed_IN_ vs Speed_OUT_) (*F*(1,7017.0) = 144.761 *p* < 0.001, *d* = 0.29): Speed_OUT_ were faster than Speed_IN_ (*z* = 12.03, *p* < 0.0001, *d* = 0.14). A significant effect was observed for the rivalry (happy vs neutral) (*F*(1,7021) = 63.288 *p* < 0.001, *d* = 0.19): *neutral* were faster than *happy* (*z* = −7.955 *p* < 0.0001, *d* = −0.09). A significant interaction was found between percepts in rivalry and the transition (*F*(1,7017.9) = 4.628 *p* = 0.031, η2 = 0.0006): Speed_IN_ for happy faces were slower than Speed_IN_ for neutral faces (*z* = −4.267 *p* < 0.001, *d* = −0.05) and Speed_OUT_ for happy faces were slower than Speed_OUT_ for neutral faces (*z* = −6.961 *p* < 0.001, *d* = −0.08). Speed_IN_ for happy faces were slower than Speed_OUT_ for happy faces (*z* = 8.06 *p* < 0.001, *d* = 0.1) and Speed_IN_ for neutral faces were faster than Speed_OUT_ for neutral faces (*z* = 8.969 *p* < 0.001, *d* = 0.11).

**FIGURE 7:**
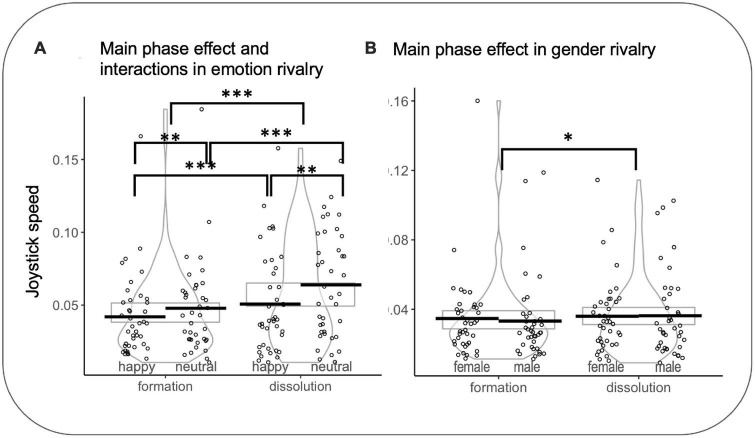
**(A)** Main effect of phase and interactions for joystick speed in emotion rivalry. **(B)** Main effect of phase for joystick speed in gender rivalry. Rectangles, beans, and points represent confidence intervals, smoothed densities within formation and dissolution, and participants’ mean data, respectively. *p<0.05, **p<0.01, ***p<0.001.

In gender rivalry, a significant effect was observed for the transition (Speed_IN_ vs Speed_OUT_) (*F*(1,7094.3) = 22.175 *p* < 0.001, *d* = 0.11): Speed_OUT_ were faster than Speed_IN_ (*z* = 1.28 *p* = 0.039, *d* = 0.06) (see Figure 7). It is important to note that, in some cases, such as in rivalry using faces, the two competing images may merge into a stable perception, eliminating binocular rivalry transitions (Klink et al., 2017). In these cases, it would be impossible to compute speed metrics.

### Questionnaires

In exploratory analyses, we tested if MP and Speed were correlated (Pearson, two-sided correlations) with the TAS-20 and the IRI. No correlations were significant with the IRI scores or TAS-20.

## Discussion

The dynamics of consciousness (from formation to dissolution) is rarely examined in consciousness research, and even when it is considered, the studies have mostly focused on the phase of formation of the conscious content. In contrast and complementary to consciousness genesis, a few researchers have drawn attention to the anti-genesis of consciousness, that is the phase in which the content fades out from consciousness (Pun et al., 2012; Aru and Bachmann, 2017). Aru and Bachmann (2017) have, for example, proposed the possibility that asymmetrical inertia could characterize the two phases of formation and dissolution of the conscious content.

One limitation in investigating the entire dynamics of consciousness is that not all paradigms allow such investigation.

The present work’s main objective was to gain information that could allow approximating the temporal evolution of consciousness hypothesized by Aru and Bachmann, in the specific context of face perception. Here, we used a binocular rivalry task to examine the time course of consciousness. In the first condition, the stimuli placed in rivalry were two different facial expressions of the same individual (neutral vs. happy); in the second condition, the rivalrous stimuli were the faces of individuals of different gender (female vs. male) but with the same expression.

We asked participants to map their conscious experience using a joystick. The movements of the joystick were then processed in such a way as to obtain some *ad hoc* variables that would provide a proxy of the formation and dissolution phases of the conscious content. In particular, the movements were analyzed in terms of the speed of the transitions in binocular rivalry in such a way as to have distinct measures for the formation phase (Speed_IN_, i.e. the joystick velocity for the formation of the dominant percept) and for the dissolution phase (Speed_OUT_, i.e. the joystick velocity for the dissolution of the dominant percept). Furthermore, we computed some traditional metrics commonly examined in binocular rivalry studies, namely mean of predominance (i.e. the average of the joystick signals) and cumulative time (i.e. the sum of the periods of stable perception). Importantly, both mean of predominance and cumulative time measures replicated the effect found with regard to happy expression in BR in which happy percept dominates over neutral percept (Alpers and Gerdes, 2007; Yoon et al., 2009).

The results supported Aru and Bachmann’s (2017) hypothesis regarding the possible different inertia between the formation and the dissolution of conscious content. Indeed, a general phase effect was found in both rivalrous conditions (i.e., emotion and gender). The formation phase was slower than the dissolution phase, indicating an asymmetric inertia of formation and dissolution of the conscious content with a slower inertia for the formation phase than the dissolution phase. Furthermore, although in the case of the gender rivalry condition, only a general effect of the phase was observed, with contents’ formation being slower than dissolution, the results of the emotion rivalry condition highlighted additional dynamics of the temporal evolution of consciousness. One potential explanation^6^ for the finding is that the decision to judge a stimulus as no longer ambiguous may be easier, as it only requires the detection of a single dissimilar feature that violates the exclusive state. In contrast, for the formation of an exclusive percept, many features and their configural processing need to align across time, which may require additional attentional resources and the engagement of different perceptual and cognitive processes.

But, in this emotion condition, in addition to the general effect of the phase reported above, differences in the time course were also observed as a function of the specific facial expression, such that faces with happy expressions were associated with lower velocity indexes than neutral faces, suggesting that both their formation and dissolution as contents of consciousness were slower than for faces with a neutral expression.

To note, the attribution of the meaning to emotional facial expressions requires the combination of multiple sources of processing by visual, limbic, and sensorimotor areas (Carr et al., 2003; Fox et al., 2009; Trautmann et al., 2009; Haxby and Giobbini, 2011; Furl et al., 2013; Johnston et al., 2013; Harris et al., 2014). From this point of view, the number of involved channels/brain regions could lead to differences in the inertia of the formation and dissolution phases.

In light of our general research objective, we propose to annex a third phase between the formation and the dissolution phases in addition to the original phases proposed by Aru and Bachmann. In fact, following the content’s formation and before it fades from consciousness, the content is stabilized in consciousness for a while (i.e., stabilization phase).

In January 2023, Skerswetat and Bex published an article describing a new method called InFoRM (Indicate-Follow-Replay-Me) to study perceptual multistability, specifically in the context of binocular rivalry for Gabor patches with different orientations. As highlighted in the Introduction, the current methods used to assess multistability have limitations. InFoRM is a more advanced method than the one proposed by us for researchers interested in capturing all potential perceptual states and it offers a continuous high-temporal resolution. In this sense, we invite readers interested in implementing a method with these characteristics to refer to the work of Skerswetat and Bex (2023). InFoRM has the specific goal of mapping in detail not only the experience of “exclusive” percepts but also the different possible experiences of mixed percepts (piecemeal/superimposition; see Figure 1 on p. 4 of Skerswetat and Bex, 2023), while we were interested in mapping the subjective experience of accessing and exiting from “exclusive” face percepts with particular characteristics (connoted by a specific emotional expression or gender). Of note, faces in binocular rivalry are much less prone to piecemeal rivalry (where piecemeal rivalry is measurable in terms of low coherence index; seem e.g., (Alais and Parker, 2006) than other, low-level, stimuli (e.g., Gabor patches), likely due to the different size of receptive fields of the high-level vs. early visual areas (Blake et al., 1992). Thus stimuli such as Gabor (used in the experiment by Skerswetat and Bex, 2023) tend to be associated with a lot of mixed/piecemeal rivalry; on the other hand, high-level stimuli (such as faces) are characterized by a high coherence index (i.e., stronger alternations from one face to another, and very little piecemeal rivalry and superimposition). For example, in one of their studies, Alais and Parker (2006) reported a coherence index for faces (face 1 vs. face 2 in rivalry) equal to 80%, therefore very high. Moreover, in our study, we implemented further precautions to favor a high coherence index: (a) the participants were accurately instructed to map the experience of precise contents—rather than single features possibly diagnostic of emotion or gender—using the leftmost/rightmost joystick positions; (b) the face stimuli, both in the condition of emotion rivalry and gender rivalry, were slightly misaligned with respect to the fixation point to favor the rivalry between faces rather than between individual features (i.e., mixed percepts); (c) the face stimuli were relatively small in size (6.5° of visual angle).

The present study has some limitations which should be mentioned.

Inverted faces are often used as a control condition in cognitive neuroscience and psychology experiments involving face perception. The main reason for using inverted faces as a control condition is to test whether the observed effects are specific to the processing of upright faces or whether they are simply a result of low-level visual features (such as luminance and contrast). We have not included a control condition with inverted faces, so one possibility is that the observed pattern of results may depend at least partially on low-level features or both low and high- level features (Yang et al., 2007). Although we believe this is unlikely since we have carefully calibrated the contrast and luminance of rivalrous faces (see Method section) and counterbalanced stimuli colors, positions and coding, future research should consider adding such control condition with inverted faces.

Secondly, another potential limitation concerns the absence of a replay condition. In the replay block, a physical replay of the binocular rivalry is encoded by the participants to compare their responses between the two visual experiences (replay vs. binocular rivalry). This control allows controlling for participants’ accuracy and compliance with coding instructions. This replay method has been implemented by Skerswetat and Bex (2023) and we recommend its use. In the present study, due to time constraints, we chose to counterbalance the joystick coding instructions to control possible difference in joystick movements due to supination and pronosupination movements.

Finally, we want to propose some feasible directions. Firstly, future studies could include neural measures that can reassure about the validity of the method we proposed here by examining the relationship between the joystick velocity metrics and the trend of neural indices associated with (faces’) consciousness (Doesburg et al., 2005, 2009; Kim and Blake, 2005; Wilcke et al., 2009; Dehaene and Changeux, 2011; Blake et al., 2014; Frässle et al., 2014; Gelbard-Sagiv et al., 2018; Cha and Blake, 2019; Hernández-Lorca et al., 2019). Secondly, one possible direction for future research is to examine the possible effects of small eye movements on rivalry, which is still debated in this field since the days of the controversy between Hering and von Helmholtz. The description of these eye movements may advance our understanding of the mechanisms underlying unstable perception and provide further insight into the neural processes involved.

Thirdly, future investigations could explore the issue of whether the three different phases of the consciousness temporal evolution are related to each other, such that the inertia or duration of one phase could predict the inertia or duration of another phase.

To conclude, the similarity between the temporal evolution of consciousness hypothesized by Aru and Bachmann and the time course detected through the joystick in this study is remarkable, which is why we believe - with cautious optimism - that this approach can allow researchers to map with a good approximation the dynamics of the subjective experience of faces’ contents in consciousness.

## Data availability statement

The datasets presented in this study can be found in online repositories. The names of the repository/repositories and accession number(s) can be found in the article/Supplementary material.

## Ethics statement

The studies involving human participants were reviewed and approved by Ethical Board of Psychology – University of Padova. The patients/participants provided their written informed consent to participate in this study.

## Author contributions

TQ developed the study concept, programmed the experiment and prepared the stimuli, and performed the data analysis. ND gathered the data. PS, TQ, and NT interpreted the data. TQ and PS drafted the manuscript. NT provided critical revision. All authors contributed to the study design, approved the final version of the manuscript.

## Footnotes

1
https://loop-impact.frontiersin.org/impact/article/238706#totalviews/views

2
https://www.kdef.se/

3
https://www.gimp.org

4
https://fiji.sc

5
https://osf.io/2pzmg/

6 We thank the reviewer for this interesting possible explanation.

## References

[B1] Alais D. Blake R. (2005). *Binocular Rivalry.* Cambridge, MA: MIT Press.

[B2] Alais D. Parker A. (2006). Independent binocular rivalry processes for motion and form. *Neuron* 52 911–920. 10.1016/j.neuron.2006.10.027 17145510

[B3] Alpers G. Gerdes A. (2007). Here is looking at you: Emotional faces predominate in binocular rivalry. *Emotion* 7 495–506. 10.1037/1528-3542.7.3.495 17683206

[B4] Aru J. Bachmann T. (2017). In and out of consciousness: How does conscious processing (D)evolve over time? *Front. Psychol.* 8:128. 10.3389/fpsyg.2017.00128 28210236 PMC5288355

[B5] Bachmann T. (2000). *Microgenetic Approach to the Conscious Mind.* Amsterdam: John Benjamins Publishing Company. 10.1075/aicr.25

[B6] Bannerman R. Milders M. De Gelder B. Sahraie A. (2008). Influence of emotional facial expressions on binocular rivalry. *Ophthalmic Physiol. Opt*. 28 317–326. 10.1111/j.1475-1313.2008.00568.x 18565087

[B7] Bates D. Mächler M. Bolker B. Walker S. (2014). Fitting linear mixed-effects models using lme4. *arXiv.* Available online at: http://arxiv.org/abs/1406.5823 (accessed June 23, 2014).

[B8] Blake R. Brascamp J. Heeger D. (2014). Can binocular rivalry reveal neural correlates of consciousness? *Philos. Trans. R. Soc. Lond. B Biol. Sci*. 369:20130211. 10.1098/rstb.2013.0211 24639582 PMC3965165

[B9] Blake R. O’Shea R. Mueller T. (1992). Spatial zones of binocular rivalry in central and peripheral vision. *Vis. Neurosci.* 8 469–478. 10.1017/s0952523800004971 1586647

[B10] Brascamp J. Klink P. Levelt W. (2015). The ‘laws’ of binocular rivalry: 50 years of Levelt’s propositions. *Vis. Res.* 109(Pt A), 20–37. 10.1016/j.visres.2015.02.019 25749677

[B11] Carr L. Iacoboni M. Dubeau M. Mazziotta J. Lenzi G. (2003). Neural mechanisms of empathy in humans: A relay from neural systems for imitation to limbic areas. *Proc. Natl. Acad. Sci. U. S. A.* 100 5497–5502. 10.1073/pnas.0935845100 12682281 PMC154373

[B12] Carter O. Hasler F. Pettigrew J. Wallis G. Liu G. Vollenweider F. (2007). Psilocybin links binocular rivalry switch rate to attention and subjective arousal levels in humans. *Psychopharmacology* 195 415–424. 10.1007/s00213-007-0930-9 17874073

[B13] Cha O. Blake R. (2019). Evidence for neural rhythms embedded within binocular rivalry. *Proc. Natl. Acad. Sci. U. S. A.* 116 14811–14812. 10.1073/pnas.1905174116 31285320 PMC6660745

[B14] Champely S. Ekstrom C. Dalgaard P. Gill J. Weibelzahl S. Anandkumar A. (2017). *Pwr: Basic Functions for Power Analysis.* Available online at: https://nyuscholars.nyu.edu/en/publications/pwr-basic-functions-for-power-analysis (accessed March 17, 2020).

[B15] Davidson M. Alais D. van Boxtel J. Tsuchiya N. (2018). Attention periodically samples competing stimuli during binocular rivalry. *Elife* 7:e40868. 10.7554/eLife.40868 30507378 PMC6298779

[B16] Dehaene S. Changeux J. (2011). Experimental and theoretical approaches to conscious processing. *Neuron* 70 200–227. 10.1016/j.neuron.2011.03.018 21521609

[B17] Doesburg S. Green J. McDonald J. Ward L. (2009). Rhythms of consciousness: Binocular rivalry reveals large-scale oscillatory network dynamics mediating visual perception. *PLoS One* 4:e6142. 10.1371/journal.pone.0006142 19582165 PMC2702101

[B18] Doesburg S. Kitajo K. Ward L. (2005). Increased gamma-band synchrony precedes switching of conscious perceptual objects in binocular rivalry. *Neuroreport* 16 1139–1142. 10.1097/00001756-200508010-00001 16012336

[B19] Fahle M. Stemmler T. Spang K. (2011). How much of the “Unconscious” is just pre - threshold? *Front. Hum. Neurosci.* 5:120. 10.3389/fnhum.2011.00120 22025912 PMC3198031

[B20] Fox C. Iaria G. Barton J. (2009). Defining the face processing network: Optimization of the functional localizer in fMRI. *Hum. Brain Mapp*. 30 1637–1651. 10.1002/hbm.20630 18661501 PMC6870735

[B21] Frässle S. Sommer J. Jansen A. Naber M. Einhäuser W. (2014). Binocular rivalry: Frontal activity relates to introspection and action but not to perception. *J. Neurosci.* 34 1738–1747. 10.1523/JNEUROSCI.4403-13.2014 24478356 PMC6827584

[B22] Furl N. Henson R. Friston K. Calder A. (2013). Top-down control of visual responses to fear by the amygdala. *J. Neurosci.* 33 17435–17443. 10.1523/JNEUROSCI.2992-13.2013 24174677 PMC6618361

[B23] Gelbard-Sagiv H. Mudrik L. Hill M. Koch C. Fried I. (2018). Human single neuron activity precedes emergence of conscious perception. *Nat. Commun.* 9:2057. 10.1038/s41467-018-03749-0 29802308 PMC5970215

[B24] Harris R. Young A. Andrews T. (2014). Dynamic stimuli demonstrate a categorical representation of facial expression in the amygdala. *Neuropsychologia* 56 47–52. 10.1016/j.neuropsychologia.2014.01.005 24447769 PMC3988993

[B25] Haxby J. Giobbini M. I. (2011). “Distributed neural systems for face perception,” in *Oxford Handbook of Face Perception*, eds Rhodes G. Calder A. Johnson M. Haxby J. (Oxford: Oxford University Press), 93–110.

[B26] Hernández-Lorca M. Sandberg K. Kessel D. Fernández-Folgueiras U. Overgaard M. Carretié L. (2019). Binocular rivalry and emotion: Implications for neural correlates of consciousness and emotional biases in conscious perception. *Cortex* 120 539–555. 10.1016/j.cortex.2019.08.003 31521914

[B27] Hodges W. F. Fox R. (1965). Effect of arousal and intelligence on binocular rivalry rate. *Percept. Motor Skills* 20 71–75. 14286560 10.2466/pms.1965.20.1.71

[B28] Johnston P. Mayes A. Hughes M. Young A. (2013). Brain networks subserving the evaluation of static and dynamic facial expressions. *Cortex* 49 2462–2472. 10.1016/j.cortex.2013.01.002 23410736

[B29] Kim C. Blake R. (2005). Psychophysical magic: Rendering the visible ‘invisible’. *Trends Cogn. Sci*. 9 381–388. 10.1016/j.tics.2005.06.012 16006172

[B30] Klink P. Boucherie D. Denys D. Roelfsema P. Self M. (2017). Interocularly merged face percepts eliminate binocular rivalry. *Sci. Rep.* 7:7585. 10.1038/s41598-017-08023-9 28790394 PMC5548737

[B31] Klink P. Brascamp J. Blake R. van Wezel R. (2010). Experience-driven plasticity in binocular vision. *Curr. Biol.* 20 1464–1469. 10.1016/j.cub.2010.06.057 20674360 PMC2926173

[B32] Knapen T. Brascamp J. Pearson J. van Ee R. Blake R. (2011). The role of frontal and parietal brain areas in bistable perception. *J. Neurosci.* 31 10293–10301. 10.1523/JNEUROSCI.1727-11.2011 21753006 PMC3146344

[B33] Lee S. Blake R. Heeger D. (2005). Traveling waves of activity in primary visual cortex during binocular rivalry. *Nat. Neurosci.* 8 22–23. 10.1038/nn1365 15580269 PMC1215464

[B34] Maddaluno O. Aiello E. Roncoroni C. Prunas A. Bolognini N. (2022). The reading the mind in the eyes test, iowa gambling task and interpersonal reactivity index: Normative data in an italian population sample. *Arch. Clin. Neuropsychol.* 37 929–938. 10.1093/arclin/acab100 35107132

[B35] Naber M. Frässle S. Einhäuser W. (2011). Perceptual rivalry: Reflexes reveal the gradual nature of visual awareness. *PLoS One* 6:e20910. 10.1371/journal.pone.0020910 21677786 PMC3109001

[B36] Nemiah J. C. Freyberger H. Sifneos P. E. (1976). “Alexithymia: A view of the psychosomatic process,” in *Modern Trends in Psychosomatic Medicine*, Vol. 3 ed. Hill O. W. (London: Butterworths), 430–439.

[B37] Parker J. Taylor G. Bagby R. (2003). The 20-Item Toronto Alexithymia Scale. III. Reliability and factorial validity in a community population. *J. Psychosom. Res.* 55 269–275. 10.1016/s0022-3999(02)00578-0 12932802

[B38] Pun C. Emrich S. Wilson K. Stergiopoulos E. Ferber S. (2012). In and out of consciousness: Sustained electrophysiological activity reflects individual differences in perceptual awareness. *Psychon. Bull. Rev.* 19 429–435. 10.3758/s13423-012-0220-3 22297436

[B39] Quettier T. Gambarota F. Tsuchiya N. Sessa P. (2021). Blocking facial mimicry during binocular rivalry modulates visual awareness of faces with a neutral expression. *Sci. Rep.* 11:9972. 10.1038/s41598-021-89355-5 33976281 PMC8113223

[B40] R Core Team (2012). *R: A language and environment for statistical computing*. Vienna, Austria: R Foundation for Statistical Computing. Available online at: https://www.R-project.org/

[B41] Skerswetat J. Bex P. (2023). InFoRM (Indicate-Follow-Replay-Me): A novel method to measure perceptual multistability dynamics using continuous data tracking and validated estimates of visual introspection. *Conscious Cogn.* 107:103437. 10.1016/j.concog.2022.103437 36450218 PMC9840704

[B42] Skerswetat J. Formankiewicz M. Waugh S. (2018). More superimposition for contrast-modulated than luminance-modulated stimuli during binocular rivalry. *Vis. Res.* 142 40–51. 10.1016/j.visres.2017.10.002 29102622

[B43] Trautmann S. Fehr T. Herrmann M. (2009). Emotions in motion: Dynamic compared to static facial expressions of disgust and happiness reveal more widespread emotion-specific activations. *Brain Res*. 1284 100–115. 10.1016/j.brainres.2009.05.075 19501062

[B44] Wheatstone C. (1843). Contributions to the physiology of vision.—part the first. On some remarkable and hitherto unobserved phenomena of binocular vision. *Abstracts Papers Print. Philos. Transact. R. Soc. Lond.* 4 76–77.

[B45] Wilcke J. O’Shea R. Watts R. (2009). Frontoparietal activity and its structural connectivity in binocular rivalry. *Brain Res.* 1305 96–107. 10.1016/j.brainres.2009.09.080 19782667

[B46] Yang E. Zald D. Blake R. (2007). Fearful expressions gain preferential access to awareness during continuous flash suppression. *Emotion* 7 882–886. 10.1037/1528-3542.7.4.882 18039058 PMC4038625

[B47] Yang Y. Rose D. Blake R. (1992). On the variety of percepts associated with dichoptic viewing of dissimilar monocular stimuli. *Perception* 21 47–62. 10.1068/p210047 1528703

[B48] Yoon K. Hong S. Joormann J. Kang P. (2009). Perception of facial expressions of emotion during binocular rivalry. *Emotion* 9 172–182. 10.1037/a0014714 19348530

[B49] Zaki J. Ochsner K. (2012). The neuroscience of empathy: Progress, pitfalls and promise. *Nat. Neurosci.* 15 675–680. 10.1038/nn.3085 22504346

