# Supplementary material for: INs and OUTs of faces in consciousness: a study of the temporal evolution of consciousness of faces during binocular rivalry
Source: Front Hum Neurosci. 2023 May 22;17:1145653. doi: 10.3389/fnhum.2023.1145653 (PMC10241245; doi:10.3389/fnhum.2023.1145653)
Supplement: Supplementary file 1 [file Data_Sheet_1.PDF]

Supplementary Materials

Contents

1 General approach **3**

1.1 model selection 4

1.1.1 Model selection : Emotion rivalry (Speed) 4

1.1.2 Model selection : Gender rivalry (Speed) 4

1.1.3 Model selection : Emotion stability (Cumulative time) 5

1.1.4 Model selection : gender stability (cumulative time) 5

1 General approach

We used R (R Core Team, 2012) and lme4 (Bates, Maechler & Bolker, 2012) to perform a linear mixed effects analysis of the relationship between rivalry and consciousness phases. As fixed effects, we entered rivalry and phases (with interaction term) into the model. As random effects, we had intercepts for subjects. Visual inspection of residual plots did not reveal any obvious deviations from homoscedasticity or normality. P-values were obtained by anova of the full models for gender blocks and emotion blocks:

```
fit <- lmer(mean ~ rivalry*phase + (1|subject)
```

1.1 model selection

The logic of the model selection is to compare the likelihood of different models. First, the model without any factor (the null model), then each model add a factor that we are interested in.

```
model1<-lmer(speed ~ 1 + (1|subject), dat) # null model

model2<-lmer(speed ~ phase + (1|subject), dat) # add consciousness phases: formation vs dissolution

model3<-lmer(speed ~ phase + rivalry + (1|subject), dat) # add rivalry: emotion(happy vs neutral) OR ge

model4<-lmer(speed ~ rivalry*phase + (1|subject), dat) # add interaction


anova(model1,model2,model3,model4)

# Stabilisation

model1<-lmer(STB ~ 1 + (1|subject), dat) # null model

model2<-lmer(STB ~ rivalry + (1|subject), dat) # add consciousness rivalry: emotion(happy vs neutral) O

anova(model1,model2)
```

1.1.1 Model selection : Emotion rivalry (Speed)

term	npar	AIC	BIC	logLik	deviance	statistic	df	p.value
model1	3.000	-24,875.386	-24,854.800	12,440.693	-24,881.386			
model2	4.000	-25,014.287	-24,986.839	12,511.144	-25,022.287	140.901	1.000	0.000
model3	5.000	-25,073.504	-25,039.193	12,541.752	-25,083.504	61.216	1.000	0.000
model4	6.000	-25,076.132	-25,034.959	12,544.066	-25,088.132	4.628	1.000	0.031

1.1.2 Model selection : Gender rivalry (Speed)

term	npar	AIC	BIC	logLik	deviance	statistic	df	p.value
model1	3.000	-28,239.352	-28,218.735	14,122.676	-28,245.352			
model2	4.000	-28,259.495	-28,232.004	14,133.747	-28,267.495	22.142	1.000	0.000
model3	5.000	-28,257.918	-28,223.555	14,133.959	-28,267.918	0.424	1.000	0.515
model4	6.000	-28,258.001	-28,216.765	14,135.000	-28,270.001	2.083	1.000	0.149

1.1.3 Model selection : Emotion stability (Cumulative time)

term	npar	AIC	BIC	logLik	deviance	statistic	df	p.value
model1	3.000	12,729.181	12,742.657	-6,361.590	12,723.181			
model2	4.000	12,414.950	12,432.919	-6,203.475	12,406.950	316.230	1.000	0.000

1.1.4 Model selection : gender stability (cumulative time)

term	npar	AIC	BIC	logLik	deviance	statistic	df	p.value
model1	3.000	14,051.589	14,065.441	-7,022.794	14,045.589			
model2	4.000	14,050.534	14,069.003	-7,021.267	14,042.534	3.055	1.000	0.080

References

Bates, Douglas, Martin Mächler, Ben Bolker, and Steve Walker. 2014. "Fitting Linear Mixed-Effects Models Using lme4." arXiv [stat.CO]. arXiv. http://arxiv.org/abs/1406.5823.
